# The Role and Potential Pathogenic Mechanism of Particulate Matter in Childhood Asthma: A Review and Perspective

**DOI:** 10.1155/2020/8254909

**Published:** 2020-01-17

**Authors:** Xuchen Xu, Jianing Zhang, Xin Yang, Yuanyuan Zhang, Zhimin Chen

**Affiliations:** ^1^Department of Pulmonology, Children's Hospital, Zhejiang University School of Medicine, Hangzhou, China; ^2^National Clinical Research Center for Child Health, China; ^3^State Key Laboratory of Ophthalmology, Zhongshan Ophthalmic Center, Sun Yat-sen University, Guangzhou, China

## Abstract

Asthma, the most common chronic respiratory disease in children, affects numerous people worldwide. Accumulating evidence suggests that exposure to high levels of particulate matter (PM), either acutely or chronically, is associated with the exacerbation and incidence of pediatric asthma. However, the detailed pathogenic mechanisms by which PM contributes to the incidence of asthma remain largely unknown. In this short review, we summarize studies of relationships between PM and pediatric asthma and recent advances on the fundamental mechanisms of PM-related asthma, with emphases on cell death regulation and immune system responses. We further discuss the inadequacy of current studies and give a perspective on the prevention strategies for pediatric asthma.

## 1. Introduction

Asthma is the most common chronic respiratory disease in children, which is characterized by chronic airway inflammation, reversible airway obstruction, airway remodeling, increased mucus production, and airway hyperresponsiveness [1]. It currently affects approximately 334 million people and 10% of children worldwide [2, 3]. Epidemiological studies strongly suggest that the worldwide prevalence of childhood asthma is rising as a result of air pollution, especially exposure to particulate matter [4]. Particulate matter, also referred to as particle pollution or PM, is a complicated mixture of components, including nitrates, sulfates, elemental and organic carbon, organic compounds (e.g., polycyclic aromatic hydrocarbons), biological compounds (e.g., endotoxin and cell fragments), and metals (e.g., iron, copper, nickel, zinc, and vanadium) [5]. A growing body of evidence shows that high levels of particulate matter (i.e., diesel exhaust particles), ozone, sulfur dioxide, and nitrous oxide (O_3_, SO_2_, and NO_2_) are major airborne allergens enhancing the risk of atopic sensitization and exacerbation of asthma and asthma-like symptoms, which may increase the hospitalization rate, and/or mortality rate of asthma, especially in children [6, 7]. Despite the many epidemiological and clinical studies focused on the possible links between PM and childhood asthma, summary studies on the molecular pathogenesis of asthma are few.

This review gives an overview of the etiology and the fundamental molecular mechanisms between PM and pediatric asthma and propose new potential effective prevention strategies for pediatric asthma.

## 2. The Impact of PM on Asthma in Children

Regulated pollutants are carbon monoxide (CO), lead, nitric dioxide (NO_2_), ozone (O_3_), sulfur dioxide (SO_2_), and particulate matter (PM). Transition metals, polycyclic aromatic hydrocarbons, and environmentally persistent free radicals are constituents of PM, which may trigger many of the phenotypic changes associated with asthma [8, 9]. PM can be described by its “aerodynamic equivalent diameter” (AED), and particles of the same AED are likely to have the same settling velocity. PM is categorized into 4 classes by its AED: PM_10_ (smaller than 10 *μ*m), coarse PM (ranging from 2.5 to 10 *μ*m), PM_2.5_ (smaller than 2.5 *μ*m), and ultrafine PM (UFP, smaller than 0.1 *μ*m) [10]. Different sizes of PM may trigger different symptoms [11]. Accumulating evidence suggests that exposure to high levels of PM, either acutely or chronically, is associated with increased hospitalization rate, disease incidence, loss of lung function, and exacerbation of certain chronic respiratory diseases [12, 13].

Several cohort studies have demonstrated a positive relationship between increased PM and asthma incidence, especially in children. In 1993, Wjst et al. [14] firstly proposed that exposure to car exhaust fumes increased the cumulative prevalence of recurrent dyspnea and lifetime prevalence of childhood asthma. Other reports have shown that in 10 European cities, 14% of all asthma cases in children and 15% of all exacerbation of childhood asthma were attributed to exposure to air pollutants related to road traffic [15]. The periodic exposure to PM has been confirmed to play an important role in the incidence of asthma. Several studies have found that prenatal particulate matter exposure affected the incidence of childhood asthma [16–19]. For instance, Yang et al. have demonstrated that prenatal exposure to PM_10_ was found to play significant effects on airway hyperresponsiveness (AHR) (aOR 1.694, 95% CI 1.298-2.209) and was associated with the risk of a new diagnosis of asthma in school-aged children (aOR 2.056, 95% CI 1.240-3.409) [16]. In the United States, researchers also found that higher prenatal exposure to PM during 16-25 weeks of gestation is associated with early childhood asthma development and the association exists only for boys through sex-stratified analyses [17]. What's more, the relationship between exposures at the current address were less statistically significant than associations with exposures at the birth address [17, 18]. A population-based birth cohort study suggested that exposure to NO_2_ and PM_2.5_ at the birth address were consistently associated with increased asthma incidence and prevalence after age 4 years. Jung et al. also have reported that both prenatal and postnatal exposures to PM_2.5_ were associated with later development of asthma. The vulnerable time windows might be within early gestation and midgestation and infancy [19].

Another important risk in the incidence of pediatric asthma is the composition of PM. Authors have suggested that perinatal exposure to UFPs during a critical period of lung development was linked to the onset of asthma in children, independent of PM_2.5_ and NO_2_ [20]. However, an investigation from Taiwan determined that particulate matter ≤ 2.5 *μ*m (PM_2.5_), particulate matter ≤ 10 *μ*m (PM_10_), sulfur dioxide (SO_2_), and nitrogen dioxide (NO_2_) were positively associated with childhood asthma hospitalization rate, while ozone (O_3_) was negatively associated with childhood asthma hospitalization rate. In addition, SO_2_ turned to be the most significant risk factor [21]. Lovinsky-Desir et al. from New York also drew an almost same conclusion. Their study suggested that higher levels of all air pollutants (including annual NO_2_, PM_2.5_, and elemental carbon (EC); summer average O_3_; and winter average SO_2_) except for O_3_ were detected in high-asthma-prevalence neighborhoods than in lower ones, which may also indirectly lead to a rise of children asthma in those regions [22].

## 3. Mechanism of PM in Promoting Incidence of Pediatric Asthma

The potential connection between PM and pediatric asthma onset is still not fully understood, especially how PM influences the children asthma even at the gestation stages. Putting this aside, however, it is widely believed that certain cell types and signal pathways play critical roles in PM-associated pathogenesis. Cells such as epithelial cells, macrophages, and T cells and signal pathways like the nuclear factor-*κ*B signaling pathway and the EGFR-PI3K*α*-AKT/ERK pathway were also reported to be involved in the incidence of asthma.

### 3.1. Inflammation and Regulated Cell Death

The contribution of inflammation to the pathogenesis of PM-related respiratory diseases through epithelial cells, monocytes, and other immune cells has already been illustrated in Xu et al.'s paper [23]. Meanwhile, authors have investigated that PM increased the expression of amphiregulin (AREG) in human bronchial epithelial cells (HBECs) and further induced inflammation and mucus hypersecretion via the EGFR-PI3K*α*-AKT/ERK pathway, which is known to promote the development and exacerbation of asthma [24]. Similarly, it has been demonstrated that diesel exhaust particulates (DEP) augmented the pulmonary expression of CXC chemokines, followed by an increase in the number of neutrophils in the airways. Through the augmenter of KC (keratinocyte-derived chemokine) and MIP-2 (macrophage inflammatory protein-2), the asthma-like inflammation, including phenotypic changes like increased inflammatory cells, mucin production, and AHR [25], could be significantly deteriorated. Moreover, accumulating literature investigated the role of PM in the inducement of reactive oxygen species (ROS) and epithelial cell apoptosis [26–28]. On one hand, ROS activates the promoters of cytokines and chemokines involved in allergic inflammation through activator protein-1 and nuclear factor-*κ*B signaling pathways, which may explain the exacerbation of allergic inflammation [29–33]. On the other hand, the apoptosis of epithelial cells may contribute to the promotion of epithelial shedding by activating not only the tumor necrosis factor-alpha- (TNF-*α*-) induced pathways, but also the mitochondrial pathways, which regulate the airway reflexes in asthma [34].

In addition to apoptosis, other forms of regulated cell death including autophagy and necroptosis have also been reported to play a vital role in the incidence and exacerbation of PM-related asthma [35]. It has been reported that PM exposure inactivated MTOR (mechanistic target of rapamycin kinase) and then enhanced autophagy via the TSC2 (TSC complex subunit 2) pathway in HBE cells and in mouse airway epithelium [36, 37]. In epithelial cells, autophagy activities may serve as a functional response to noxious or inflammatory signals such as interleukin- (IL-) 13, a Th2-type pleiotropic cytokine for which the level of abundance is increased in asthma [38]. What's more, IL-13 promotes transforming growth factor-b1- (TGF-b1-) dependent airway remodeling through the proliferation of subepithelial mesenchymal cells, which along with other proinflammatory cells, produces a thickened subepithelial layer which is considered to be a distinct feature of severe asthma. Thus, PM may induce autophagy through the response to IL-13 promoting TGF-b1 airway remodeling and the loss of lung function in asthma [39]. Furthermore, autophagy is associated with many of the chemical-caused cytotoxic mechanisms, including mitochondrial dysfunction, oxidative stress, and inflammation mentioned above which contribute to the exacerbation of asthma [37].

Another report has found that BALB/c mice exposed to PM_2.5_ significantly displayed increased neutrophils in bronchoalveolar lavage fluid (BALF) and bronchitis. Alveolar epithelial hyperplasia and necroptosis have also been observed which were indicated by increased TUNEL, Fas, Receptor-interacting protein 3 (RIPK3), and MLKL measure. Meanwhile, necroptosis, as a direct mechanism for IL-33 release, may have major implications in type 2 immune responses and contribute to the development of allergic airway disease [40, 41].

### 3.2. Immunity

#### 3.2.1. Innate Immunity

There is a growing interest in the potential roles of macrophages in disease progression. Airway macrophages (AMs) could react quickly to the inhaled PM as an initial barrier. Phagocytosis of inhaled carbonaceous PM by AMs is impaired in severe asthma [42]. DEPs could induce macrophage activation, which leads to cytokine production as well as facilitates allergen presentation to type 2 T-helper lymphocytes. The T-helper 2 cell is crucial for the inducement of IgE isotype switching in B lymphocytes which may enhance allergic inflammation as well as bronchial hyperreactivity [43, 44]. Moreover, macrophage polarization has been shown to have a profound impact on asthma pathogenesis. Authors have found that PM_2.5_ exposure increased the expression of proinflammatory cytokines and enhanced inflammatory M1 polarization through the ROS pathway in primary mouse peritoneal macrophages and Raw 264.7 cells [45, 46]. Meanwhile, a report used two distinct mouse models to identify the M1 or M2 cells: house dust mite- (HDM-) induced allergic asthma model and farm dust extract- (FDE-) induced nonallergic asthma. The FDE model showed M1 polarization with increased expression of Th1 and Th17 cells which may raise the possibility that M1 cells were the major effector macrophages in nonallergic asthma [47]. Nevertheless, PM could induce M2 polarization in a particulate situation. Thevenot et al. have investigated that chronic alcohol induced M2 polarization enhancing pulmonary disease caused by PM. In the alcoholic lung, the effects of PM exposure to macrophages led to the latent TGF-*β* release accompanied by M2 polarization of AMs [48]. M2-polarized macrophages can be further divided into three subpopulations, M2a, M2b, and M2c, according to specific stimulators [49]. M2a cells secrete high levels of IL-13 and chemokines including chemokine (C-C motif) ligand- (CCL-) 17, CCL-18, CCL-22, and CCL-24, which activate Th2 cells and promote eosinophil infiltration into the lungs [45].

Similar mechanisms in epithelial cells including ROS generation and mitochondria-mediated apoptotic pathways could be well applied in alveolar macrophages [50]. Macrophages also undergo necrosis which may interfere with the phagocytosis and the expression of many genes that participate in inflammatory reactions in the lung [51].

Residual Oil Fly Ash (ROFA), a complex mixture of sulfates, carbon- and nitrogen-containing compounds, and metals (primarily vanadium), has been shown to enhance allergen-induced pulmonary allergic response, with significant elevations in allergen-mediated eosinophilia, airway epithelial remodeling, and AHR [52, 53]. Toll-like receptors (TLRs) are protective immune sentries that sense pathogen-associated molecular patterns (PAMPs) such as unmethylated double-stranded DNA (CpG), single-stranded RNA (ssRNA), lipoproteins, lipopolysaccharide (LPS), and flagellin. In innate immune myeloid cells, TLRs induce the secretion of inflammatory cytokines, thereby engaging lymphocytes to mount an adaptive, antigen-specific immune response that ultimately eradicates the invading microbes [54]. Interestingly, recent literature has demonstrated a new mechanism of vanadate's (a component of ROFA) effect through augmenting the function of Toll-like receptor 4 (TLR4) by suppressing TLR4 degradation. Increased mRNA expression of TLR4 was observed in neutrophilic asthma. Therefore, ROFA's surface metals, especially vanadium, may be the key determinants in the induction and/or amplification of allergic responses [55, 56].

#### 3.2.2. Acquired Immunity

As crucial mediators between innate and acquired immunity, dendritic cells (DCs) play an important role in both the induction and exacerbation of PM-related asthma. Exposure to PM could not only alter the maturation but also the activation of DCs. Ferry et al. have demonstrated that the number of DCs was increased accompanied by the maturation status of pulmonary DCs, enlarged mediastinal lymph nodes after PM exposure, as evidenced by the increased expression of CD80 and CD86. CD80 and CD86 expression was associated with T helper response which might reflect the ability of DCs to induce Th1/Th2 immune responses [57, 58]. In addition, studies in vitro have investigated that ROFA directly induced the maturation of DCs upregulating the expression of costimulatory molecules and cytokines and matrix metalloproteinase (MMP) production via an up-dependent and oxidative stress-dependent pattern. Transferring ROFA-treated bone marrow-derived DCs (BMDCs) to allergic mice could exacerbate eosinophilic inflammation and increase epithelial and goblet cells [59]. Meanwhile, in ovalbumin- (OVA-) sensitized BMDCs, PM_2.5_ could enhance DC activation as evidenced by the increased expression of IL-1*β*, CD80, CD86, and MHC-II [60]. PM also acts on human epithelial cells promoting thymic stromal lymphopoietin (TSLP) release which polarized the DCs towards a Th2 immune response [61]. Certainly, PM-exposed BMDCs exhibit a very heterogeneous immunological activation which depends on the type, element, and dose of the particle [62]. The transcription factor nuclear factor (erythroid-derived 2)-like 2 (Nrf2) has reportedly been associated with the pathological process of PM-related asthma. Nrf2 deficiency in DCs may enhance the adjuvant effect of UFP on allergic sensitization through the change of immune-polarizing cytokine milieu [63].

Since DCs have been reported to play a key role in promoting the exacerbation of PM-related asthma, the function of T cells could not be ignored. Previous studies have demonstrated that instillation of PM combined with HDM sensitization in vivo, could induce Th17/Th2 coexpressing cells [64, 65]. Meanwhile, data has shown that UPM not only functions like an adjuvant but also could be a source of antigen stimulating the generation of Th1, Th2, and Th17 effectors, which may aggravate the symptom of asthma [66]. Similarly, Acciani et al. have investigated that the exposure of young mice to DEP exacerbated allergic responses characteristic of more severe asthma phenotypes, including the increase of Th17 effector T cells, eosinophils, and dual-positive (Th2/Th17) effector T cells, as well as increases in IgE, IL-17A, and Th2 cytokines [67]. Furthermore, PM could induce TLR signaling, especially TLR2 and TLR4 signaling, to trigger Th2-dominant lung allergic inflammation via the MyD88-dependent pathway [68]. It also could promote the incidence and development of asthma via the upregulated expression of IL-7 and other relevant inflammatory factors (such as IL-4, IL-8, and IL-17) and change the equilibrium between Treg and Th17 cells [69]. Interestingly, different size-segregated PM may trigger different immune responses in mice. Authors from Taiwan collected PM_10_, PM_2.5_, PM_1_, and PM_0.1_ samples into the investigation. They found that only exposure to PM_10_ could induce inflammatory responses and allergic immune response as evidence by the recruitment of cells (neutrophils and eosinophils) and the upregulated release of Th1-related cytokines (TNF-*α* and IFN-*γ*) and Th2-related cytokines (IL-5 and IL-13) [70].

Nevertheless, another report has found that early-life exposure to PM could also cause pulmonary immunosuppression. They observed that infant mice exposed to PM+HDM failed to develop a typical asthma phenotype including airway hyperresponsiveness, Th2 inflammation, Muc5ac expression, eosinophilia, and HDM-specific Ig compared to only HDM-exposed mice. They further confirmed that early-life PM exposure induced an immunosuppressive environment and the increase of tolerogenic DCs and Tregs, which led to the suppression of Th2 responses. Despite having early-life immunosuppression, the mice would develop severe allergic inflammation after a challenge with allergen in adulthood [71]. A similar phenomenon could be identified while infant mice were exposed to PM in utero. In the C57BL/6 mice, offspring exposed in utero to filtered air (FA) and challenged with HDM exhibited a robust response in inflammatory cytokines IL-13 and IL-17. In contrast, this response was lost in offspring exposed to PM in utero. Circulating IL-10 was significantly upregulated in C57BL/6 offspring exposed to PM, suggesting increased regulatory T cell expression and suppressed Th2/Th17 response [72]. Meanwhile, Wang et al. have reported that maternal exposure to PM enhanced postnatal asthma incidence in mice, which might be related to the inhibition of Th1/Th17 maturation and systemic oxidative stress [73].

Recent studies have focused on the potential importance of the aryl hydrocarbon receptor (AhR) in linking the PM exposure and the development of asthma and allergic diseases. AhR is a ligand-activated transcription factor from the Per-Arnt-Sim (PAS) superfamily and is recognized as a receptor for many of the common environmental contaminants, including polychlorinated biphenyls (PCBs) and PAHs, such as benzo(*α*)pyrene (BaP). In the case of ligand interaction, AhR in the cytoplasm translocates into the nucleus and binds to the specific regulatory DNA sequences known as dioxin response elements (DREs) located within the promoters of target genes such as CYP1A1 and CYP1B1 etc., as well as those known to be critical in immune regulation, including inflammatory (TNF-*α* and IL-6) and T-cell differentiation genes (Foxp3, IL-17, and GATA3) [74]. An investigation has shown that PM enhances DC activation that primed naïve CD4+ T cell differentiation to a Th17-like phenotype through an AhR-dependent manner [60]. Wang et al. have shown that BaP promotes dermatophagoides group 1 allergen- (Der f 1-) induced epithelial cytokine release, particularly TSLP and IL-33, and Th2 immune response through the AhR-ROS axis [75]. Meanwhile, DEP may suppress the expression of CXCL10 via AhR signaling pathways that contribute to alterations in the recruitment of CXCR3+ cells to the airway epithelium including Th1 CD4+ T-cells, which may destine the allergic airway inflammation through the potential promotion of Th2-type responses [76].

As summarized above, it is obvious that PM contributes to the incidence, development, and exacerbation of asthma phenotypes.

### 3.3. Genetic Factors

Accumulating studies have found that exposure to PM was a risk factor that may link to DNA differences in asthma. Regional DNA methylation probably was the most well-studied. Both short-term and long-term exposures to high levels of CO, NO_2_, and PM_2.5_ were associated with alterations in differentially methylated regions of Foxp3 and IL-10. Hypermethylation of CpG islands in Foxp3 associated with chronic exposure to DEP leads to the suppression of Treg function and increased asthma severity as assessed by symptoms and lung function [77]. In addition, vanadium (a component of ROFA) was associated with altered DNA methylation of allergic and proinflammatory asthma genes implicated in air-pollution-related asthma [78]. Recently, authors have demonstrated that health effects of acute particulate exposure on asthma were associated with changes in cysteinyl leukotriene (CysLTR_1_) expression and methylation of CpG sites on CysLTR_1_ and leukotriene C4 synthase genes [79]. Variant alleles of TLR2 and TLR4 genes also influenced the susceptibility to adverse effects of traffic-related air pollution on childhood asthma. It has been reported that two TLR2 single-nucleotide polymorphisms (SNPs) and four TLR4 SNPs significantly modified the effect of air pollution on the prevalence of doctor-diagnosed asthma from birth up to 8 years of age. The risk of having doctor-diagnosed asthma increased with increasing PM_2.5_ levels in children with at least one copy of the TLR2 rs4696480 A allele (OR 2.0 (95% CI 1.2 to 3.1) for an interquartile range increase in exposure). Similar observations were present with the following TLR4 genotypes [80]. Moreover, T-cell immunoglobulin and mucin domain 1 (TIM-1) was found to be an important susceptibility gene for asthma and allergy. TIM-1 can be activated by the exposure of phosphatidylserine (PtdSer) on apoptotic cells through which asthma was further induced. In sum, PM_2.5_ can increase apoptosis and TIM-1 activation, which may increase AHR associated with allergic asthma [81].

## 4. Strategies Modify Childhood Asthma Response to PM

As numerous studies have focused on the mechanism by which PM promotes the incidence of asthma, authors recently have demonstrated some strategies to improve the impact of PM on asthma. For instance, Bose et al. have found that higher serum 25-hydroxy (25-OH) vitamin D levels could reduce the adverse respiratory response associated with indoor PM_2.5_ exposures among obese urban children with asthma [82]. Moreover, fatty acid intake, specifically Omega-3 and Omega-6, can modify asthma severity and response to indoor air pollution in children [83]. A diet high in fruits and vegetables and of antioxidant vitamin supplements has been confirmed to support an important role for oxidative stress in the pathways by which outdoor air pollution adversely affects asthma [84, 85].

## 5. Conclusion

As summarized above, the studies explore the possible link between PM and pediatric asthma and the potential mechanism mainly focuses on the regulated cell death, innate immunity, acquired immunity, and genes. These studies provide a wide range of information on the pathophysiology of PM-related asthma and, as a consequence, support the development of new strategies for modifying PM-related asthma. However, there are still some divergent aspects regarding the mechanistic research between PM and asthma: (1) The periodic exposure to PM may influence the incidence of asthma. What are the potential mechanisms when being exposed to PM in fetuses, infants, preschool children, and school-aged children? (2) Different-sized PM may trigger different cellular damages or immune responses in the exacerbation and development of asthma. What are the potential mechanisms? (3) There are no definite studies on the comparison of the differences between mechanisms of the incidence and exacerbation of childhood asthma and adult asthma. Some mechanisms mentioned above have not explicitly claimed the scope of study objects.

Although authors have demonstrated that some strategies modify asthma response to PM, such as higher vitamin D levels, fatty acid intake, and antioxidant vitamin supplements, more newly effective prevention strategies, such as the application of the autophagy inhibitor Th2/Th17-associated cytokine antagonist, should take into consideration the potential molecular pathogenic mechanisms.

In general, accumulating mechanistic studies keep on shedding new light on our understanding of PM-related asthma and will eventually benefit the children who are suffering from environmental particulate pollution.
